# Safety procedures for exercise testing in the scenario of COVID-19: a position statement of the Società Italiana Scienze Motorie e Sportive

**DOI:** 10.1007/s11332-020-00694-8

**Published:** 2020-09-11

**Authors:** Massimo Venturelli, Emiliano Cè, Mara Paneroni, Marco Guazzi, Giuseppe Lippi, Antonio Paoli, Carlo Baldari, Federico Schena, Fabio Esposito

**Affiliations:** 1 Department of Neuroscience, Biomedicine and Movement Sciences, University of Verona, Via Casorati 43, Verona, Italy; 2 Department of Internal Medicine, University of Utah, Salt Lake City, UT USA; 3 Department of Biomedical Sciences for Health (SCIBIS), University of Milan, Milan, Italy; 4 IRCCS Galeazzi Orthopaedic Institute, Via Riccardo Galeazzi, 4, 20161 Milan, Italy; 5 Istituti Clinici Scientifici Maugeri IRCCS, Respiratory Rehabilitation of the Institute of Lumezzane, Brescia, Italy; 6 Cardiology Department, University of Milano Policlinico San Donato, Milan, Italy; 7 IRCCS, Policlinico San Donato, Milan, Italy; 8 Section of Clinical Biochemistry, Department of Life and Reproduction Sciences, University of Verona, Verona, Italy; 9 Department of Biomedical Sciences, University of Padova, Padua, Italy; 10 eCampus University Novedrate, Como, Italy; 11 CeRiSM-Centro Sport Montagna e Salute, Via del Ben 5b, Rovereto, Italy

**Keywords:** Exercise test, Virus, COVID-19, Pandemic, Recommendations

## Abstract

Recent data on coronavirus disease 2019 (COVID-19) pandemic showed that the virus is mostly conveyed by respiratory droplets that are produced at high intensity especially when an infected subject coughs or sneezes. Therefore, elevated volume ventilations, usually reached during physical efforts and exercise, are a potential source of contamination. On the other hand, the lockdown period which has lasted for nearly 2 months and is actually involving several countries worldwide, obliged a large part of human population to sedentary behaviors, drastically reducing their physical activity level, and reducing their cardiopulmonary fitness. Therefore, cardiopulmonary exercise testing could be beneficial, so that a safe and well-weighted return to pre-lockdown active lifestyle can be efficiently planned. However, specific guidelines on exercise testing safety procedures in the era of COVID-19 are unavailable so far. This article is aimed to provide an overview of safety procedures for exercise testing during and after COVID-19 worldwide pandemic.

## Introduction

Exercise testing is a well-established procedure, that has been extensively used for several decades in clinical and sub-clinical set-up. It is beyond the scope of this manuscript to provide detailed descriptions of these procedures, because exhaustive reports are available in previous publications of the American Heart Association (AHA), European Heart Association (EHA), and the American College of Sport Medicine (ACSM), to which interested readers can be referred [1–3]. This article is instead aimed to provide an overview of safety procedures for exercise testing during and after coronavirus disease 2019 (COVID-19) worldwide pandemic.

## Introduction to the COVID-19

Data from the World Health Organization (WHO) have revealed that COVID-19 is a worldwide pandemic, which has now infected many million patients around the world and has already caused a dramatic number of deaths. Despite the number of new cases has considerably stabilized or has even declined in some countries, a large number of COVID-19 patients will now experience long-term sequels impacting both the pulmonary and cardiovascular systems [4]. SARS-CoV-2 interplays with the pulmonary cardiovascular systems on multiple levels, not only increasing morbidity in patients with lungs and cardiovascular diseases, but also potentially exacerbating many underlying dysfunctions [5]. The causal agent of COVID-19, SARS-CoV-2, enter the host cells through its natural receptor angiotensin-converting enzyme 2 (ACE2). This protein is expressed at the surface of cells of many tissues and organs, such as nose, tongue, lung, heart, gastrointestinal system, adipose tissue, vascular endothelium, liver, kidney and testis, thus accounting the multiple organ dysfunction (MOF) that can develop in patients with severe/critical SARS-CoV-2 infection [6]. The presence of mild symptoms is disproportionate to the severity of illness [7]. The most common symptoms encompass fever (98%), cough (77%), dyspnea (63.5%) and muscle and joint soreness [8]. The rationale of symptoms distribution across organs is partially explained by the distribution and expression level of ACE-2, which is particularly enriched in above-mentioned organs and tissues [5, 6].

## The role of exercise testing in asymptomatic individuals

Asymptomatic individuals often present an apparently health status, however, they could still be positive to SARS-CoV-2 and potentially being contagious, although this last point is still a matter of debate toward the medical community. The use of safety procedures, though, becomes imperative in particular when testing an asymptomatic individual [10].

The lockdown period which has lasted for nearly 2 months and is actually involving several countries worldwide, obliged a large part of human population to sedentary behaviors, drastically reducing their physical activity level, and thus possibly reducing their cardiopulmonary health [11, 12]. Therefore, cardiopulmonary exercise testing could be beneficial, so that a safe and well-weighted return to pre-lockdown active lifestyle can be efficiently planned.

## Indications and safety

Recent data on COVID-19 pandemic showed that the virus is mostly conveyed by respiratory droplets that are produced at high intensity especially when an infected subject coughs or sneezes. These droplets can be inhaled into the lungs of individuals in the close proximity (< 1 m), or can land on surfaces, where they can then be touched and transported by hand to the body [13]. Current WHO and National Centers for Disease Control and Prevention recommendations for use of personal protective equipment (PPE) are in agreement that standard, contact precautions with face mask, eye protection, gown, and gloves will be necessary. When performing procedures that encompass the risk of generating aerosol, such as exercise testing, additional PPE may be required, including controlled or powered air purifying respirators and specific face mask for the personnel in the close proximity of patients performing the test. These procedures should be followed both while testing post-COVID-19 patients most important when we test asymptomatic individuals. However, while with the former the main goal is to prevent the onset of a relapse (their possible contagious level is still a point of discussion), with asymptomatic subject (with unknown infection status) the risk is to underestimate their possible infectious.

## Measures to prevent infection by SARS-CoV-2

The subjects at higher risk of SARS-CoV-2 infectiveness are those who in close contact with COVID-19 patients, and thereby healthcare workers engaged assistance to the patients or laboratory staff engaged in handling biological samples of COVID-19 patients without recommended PPE or using unsuitable PPE (Table 1). It is hence essential that the personnel involved is properly trained and educated on the risks of work-associated risk exposure, on the available prevention and protection measures, as well as on the clinical features of COVID-19. The most effective prevention measures that shall be applied in the healthcare sector, including personnel dedicated to the exercise testing procedure, are:

- To frequently wash hand using soap or alcohol-based solution or hand sanitizing gel.
- Avoid touching eyes, nose, and mouth with the hands;
- Coughing or sneezing inside the elbow, with the arm bent or a handkerchief, preferably disposable, which must then be immediately eliminated;
- Always wear a surgical mask and perform hand hygiene after removing the mask;
- Avoid close contact by keeping the distance of at least 1 m (preferably 2 m, whenever feasible) from other people, especially those with respiratory symptoms.

**Table 1:** PPE for exercise testing procedures

Setting	Target personnel or patients	Activity	PPE equipment
Triage	Health professional/technician/physiologist	Preliminary screening (does not involve subject contact)	Disposable Gloves Surgical mask Eye/face protection^a^
CPET laboratory	Health professional/technician/physiologist	CPET	Single use disposable Gloves Disposable fluid-resistant coverall/gown Face mask respirator (FPP2/FPP3) Eye/face protection^a^ Overshoe Protective headgear
	Subject/patient	CPET	Hands/arm washing before/after Surgical mask before/after
Transit area	Health professional/lab technician/physiologist		Surgical mask
	Subject/patient		Surgical mask

*CPET* cardiopulmonary exercise test

^a^This may be single or reusable face/eye protection/full face visor or goggles

Additional precautions seem necessary, including the correct use of PPE along with appropriate education on their wearing, usage, undressing and elimination. This is especially important considering that although the leading means of SARS-CoV-2 transmission are through droplets and direct contact, specific maneuvers and other activities which may generate aerosols are also listed as potential risk procedures. Hand hygiene and appropriate use of PPE is hence always essential to prevent contact transmission.

Nevertheless, PPE are effective for protecting operators only in combination with a larger set of measures introduced among administrative procedural and technical activities for promotion of health-care setting.

In particular, in the current epidemiological scenario and with an ongoing risk of global shortage of PPE, the following WHO recommendations about the need to optimize their use shall be especially adopted:

- Ensure appropriate use of PPE;
- Ensure availability of a sufficient quantity and quality of PPE for protecting operators and people according to the specific risk of infection;
- Coordinate the management of PPE supply chain.

All the personnel involved in exercise testing procedure shall also be:

1. Appropriately trained and updated on occupational exposure risks and measures of prevention and protection;
2. Aware of the leading clinical features of COVID-19, so enabling timely identification and isolation of suspected cases;
3. Invited to participate to specific training and courses;
4. Aware of the importance of adopting standard precautions in assisting patients, with specific focus on hand hygiene before and after contacts with patients.

## Triaging subjects and visits

**Fig. 1:**
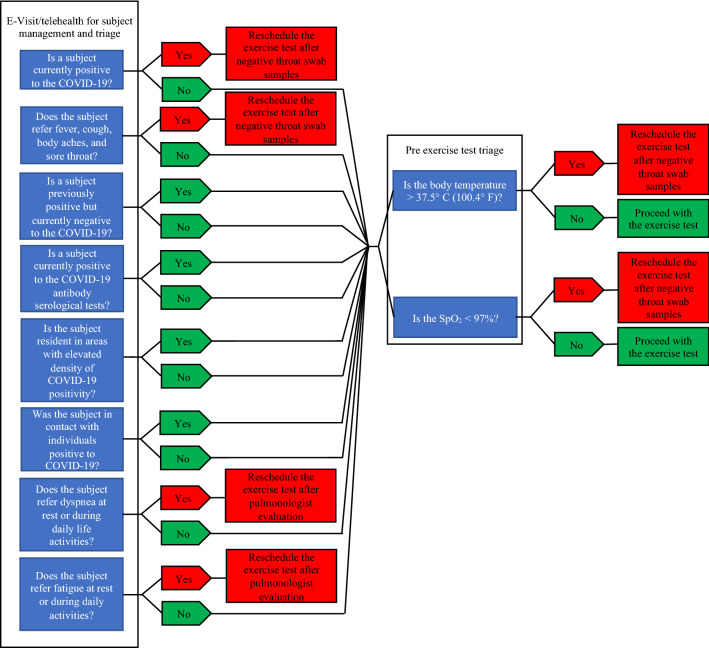
Exercise testing triage. E-visit = on-line consultation; SpO_2_ = blood oxygen saturation

The use of telemedicine remote diagnosis and treatment of patients by means of telecommunications technology is one of the leading measures to help preventing further spread of COVID-19 **(**Fig. 1). This technology, already used by various health care systems and hospitals [14], is ideal in a pandemic scenario emergencies since it limit exposure to asymptomatic (but possibly contagious) individuals. Other essential principles are minimization of direct patient–personnel interactions and number of health-care specialist required for the management of each patient to the minimum required.

## Personal protective equipment

In the current COVID-19 emergency situation, the PPE selection shall be based on the risk of SARS-CoV-2 transmission, which depends on:

- Means of transmission (especially through droplets and/or direct contact);
- Type of patient: the most contagious patients are those with cough and/or sneeze; if these patients wear a face mask or cover nose and mouth with a tissue, virus spread can be considerably lowered;
- Type of care contact—the risk increases when:
  - The contact is close (< 1 m) and prolonged (> 15 min).
  - The contact is repeated or continuous, so that it will increase the overall exposure time in the hospital and in other health care environments (such as for operators involved in repeated and/or continued medical assistance/evaluation of suspected and confirmed COVID-19 cases)
  - Performing maneuvers and procedures at high risk of producing aerosols, especially managing patient secretions.

In a context of emergencies and lack of PPE, facial filters such as FFP2 or FFP3 must be prioritized for health care professionals engaged in areas where procedures are carried out to risk of aerosol generation such as gym, recreational association, sport association or during exercise testing.

Table 1 describes the main PPEs that shall be used during exercise testing in post-COVID-19 scenario.

## Cleaning and safety procedures for the instruments

During testing, parts in direct contact with patient skin or mucosa, masks used for gas exchange analyses, even small condensation droplets or aerosols are possible contaminants. Therefore, all potentially contaminated parts must be used according to the time requested to the cleaning, as indicated by the manufacturer [15].

Since the safety of patients and staff is extremely important, the European Respiratory Society (ERS) recommend specific safety precautions while carrying out Lung function testing during COVID-19 pandemic [16]:

- Lung function testing should be performed always with a high specification disposable in-line bacterial and viral filter in place (with minimum proven efficiency for high expiratory flow of 600–700 L/min). The use of disposable combined mouthpieces/sensors is not recommended at this time. The only exception is when a filter is added.
- Maximize the use of single use consumables and dispose of the items with care e.g. nose clips, rubber mouthpieces, etc.
- Where reusable items are utilized, they should be managed carefully and should be thoroughly cleaned as recommended by local infection control policy.
  - Use filters to minimize escape of aerosol from the exhalation ports when using nebulizers.
  - During exercise testing, the use of filters connected to the inhalation/exhalation port of the facemask or mouthpiece can reduce the aerosol transmission. This could result in an increase the resistance to airflow as the ventilatory demand of exercise increases (humidification of the filter and increase of the resistance at increased ventilatory flow), therefore, rendering the results of exercise less reliable, especially in dyspneic patients. However, more work needs to be done in this area before a consensus on their use can be agreed. Thus, the use of filters during exercise testing cannot be recommended and full PPE must be worn when conducting these tests.

Re-usable parts, when properly maintained and reused according to these instructions, should be verified to function within basic specifications for a minimum of 50 cycles. Proper functioning of applicable parts after reprocessing shall be checked with both flowmeter and gas calibration, verifying that the values of all calibration factors are within acceptable range (Table 2) [17].

**Table 2:** Antimicrobial agents effective against various coronaviruses

Antimicrobial agent	Concentration (%)	Coronaviruses tested
Ethyl alcohol	70	HCoV-229E, MHV-2, MHV-N, CCV, TGEV
Sodium hypochlorite (active chlorine)	0.1–05	HCoV-229E
	0.05–0.1	SARS-CoV
Povidone-iodine	10 (1% iodine)	HCoV-229E
Glutaraldehyde	2	HCoV-229E
Isopropanol	50	MHV-2, MHV-N, CCV
Benzalkonium chloride	0.05	MHV-2, MHV-N, CCV
Sodium chlorite	0.23	MHV-2, MHV-N, CCV
Formaldehyde	0.7	MHV-2, MHV-N, CCV

Modified from “European Centre for Disease Prevention and Control. Interim guidance for environmental cleaning in non-healthcare facilities exposed to SARS-CoV-2. Stockholm: ECDC; Lehuman coronavirus

*229E* HCoV-229E, *MHV-2 and MHV-N* mouse hepatitis virus, *CCV* canine coronavirus, *TGEV* transmissible gastroenteritis virus, *SARS-CoV2* respiratory syndrome coronavirus acute severe

In preparing the devices for cardiopulmonary testing, or at the end of the test, the following general indications shall be considered:

- Assign trained and qualified staff for handing and reuse of components.
- Do not immerse the device or pour cleaning liquids over and into the device.
- To minimize the infection risk and maintain optimal performance, verify that:
  - Device and re-usable parts are cleaned and disinfected prior their first usage
  - Single-use items are disposed after each test
- Parts showing defects on material surfaces (cracks, brittleness) are disposed and standard personal protective (PPE) measures (hand hygiene, gloves, gown, mask, face shields) are always used when necessary
- Re-usable parts are disinfected immediately after use

Moreover, solutions for disinfection shall be:

- Applied thoroughly to all surfaces of the instruments needing disinfection
- Used according to the instructions and the length of contact provided by the manufacturer
- Always stored and prepared in clearly marked containers, thus preventing accidental use/misuse

### Reprocessing

Rinsing is a very important activity in the reprocessing procedure, aimed to remove organic material, lubricants, or even cleaning agents that can deactivate the chemical liquid disinfectants and thus inhibit the anti-microbial effect. Sterile water is recommended for rinsing, when available, though the use of potable tap water can also be accepted [18, 19].

### Pretreatment

The pretreatment should be completed as soon as possible after use.

When choosing a suitable detergent and disinfectant, it shall be considered that:

- Manufacturer’s safety precautions must be strictly observed, and adapted for cleaning and disinfection of metal and plastic instruments;
- The agent used has proven antimicrobial efficacy (e.g., CE marked or legally marketed in the US);
- The chemicals are compatible with re-usable parts;
- The manufacturer’s specifications on concentration and reaction time must be always respected.

### Maintenance

Disassembled components need to be reassembled after reprocessing. After cleaning and disinfection, the o-rings shall be lubricated with medical grade biocompatible Vaseline.

## Conclusions

This article is aimed to provide an overview of safety procedures for exercise testing during and after COVID-19 worldwide pandemic. The safety recommendations presented in this manuscript applied into exercise physiology laboratory, gym, sport association, etc. and integrated with updated local guidelines according to the local laws and WHO safety recommendations for the COVID-19 pandemic.
